# ANRIL Promoter DNA Methylation: A Perinatal Marker for Later Adiposity

**DOI:** 10.1016/j.ebiom.2017.03.037

**Published:** 2017-04-26

**Authors:** Karen Lillycrop, Robert Murray, Clara Cheong, Ai Ling Teh, Rebecca Clarke-Harris, Sheila Barton, Paula Costello, Emma Garratt, Eloise Cook, Philip Titcombe, Bhuvaneshwari Shunmuganathan, Samantha J. Liew, Yong-Cai Chua, Xinyi Lin, Yonghui Wu, Graham C. Burdge, Cyrus Cooper, Hazel M. Inskip, Neerja Karnani, James C. Hopkins, Caroline E. Childs, Carolina Paras Chavez, Philip C. Calder, Fabian Yap, Yung Seng Lee, Yap Seng Chong, Philip E. Melton, Lawrie Beilin, Rae-Chi Huang, Peter D. Gluckman, Nick Harvey, Mark A. Hanson, Joanna D. Holbrook, The EpiGen Consortium, Keith M. Godfrey

**Affiliations:** a Centre for Biological Sciences, Faculty of Natural and Environmental Sciences, University of Southampton, Southampton, UK; b NIHR Southampton Biomedical Research Centre, University of Southampton, University Hospital Southampton NHS Foundation Trust, Southampton, UK; c Academic Unit of Human Development and Health, Faculty of Medicine, University of Southampton, Southampton, UK; d Singapore Institute for Clinical Sciences (SICS), Agency for Science Technology and Research (A*STAR), Singapore; e MRC Lifecourse Epidemiology Unit, University of Southampton, Southampton, UK; f Academic Unit of Cancer Sciences, Faculty of Medicine, University of Southampton, Southampton, UK; g Department of Biological Sciences, Faculty of Science, King Abdulaziz University, Jeddah, Saudi Arabia; h Department of Paediatrics, KK Women's and Children's Hospital, Singapore; i Duke NUS Graduate School of Medicine, Singapore; j Lee Kong Chian School of Medicine, Nanyang Technological University, Singapore; k Department of Paediatrics, Yong Loo Lin School of Medicine, National University of Singapore, Singapore; l Department of Obstetrics and Gynaecology, Yong Loo Lin School of Medicine, National University of Singapore, Singapore; m Centre for Genetics of Health and Disease, University of Western, Australia; n Faculty of Health Science, Curtin University, Australia; o School of Medicine and Pharmacology, University of Western Australia, Australia; p Telethon Kids Institute, University of Western Australia, Perth, Australia; q Liggins Institute, University of Auckland, Auckland, New Zealand

**Keywords:** DNA methylation, Adiposity, Epigenetic

## Abstract

Experimental studies show a substantial contribution of early life environment to obesity risk through epigenetic processes. We examined inter-individual DNA methylation differences in human birth tissues associated with child's adiposity. We identified a novel association between the level of CpG methylation at birth within the promoter of the long non-coding RNA ANRIL (encoded at *CDKN2A*) and childhood adiposity at age 6-years. An association between ANRIL methylation and adiposity was also observed in three additional populations; in birth tissues from ethnically diverse neonates, in peripheral blood from adolescents, and in adipose tissue from adults. Additionally, CpG methylation was associated with ANRIL expression *in vivo*, and CpG mutagenesis *in vitro* inhibited ANRIL promoter activity. Furthermore, CpG methylation enhanced binding to an Estrogen Response Element within the ANRIL promoter. Our findings demonstrate that perinatal methylation at loci relevant to gene function may be a robust marker of later adiposity, providing substantial support for epigenetic processes in mediating long-term consequences of early life environment on human health.

## Highlights

• DNA methylation within the ANRIL promoter region was predictive of measures of adiposity in four independent cohorts.
• DNA methylation may be a marker for late gestation fetal undernutrition, followed by rapid postnatal weight gain.
• Altered DNA methylation associated with p16INK4a and p14ARF expression.
• ERα may bind at the ANRIL promoter, and *in vitro* E2 treatment stimulated expression of ANRIL, while reducing p14 expression.

The quality of the early life environment before birth can affect susceptibility to metabolic disease later in the lifecourse, with epigenetic regulation of gene function thought to be one mechanism through which early life environmental factors induce persistent phenotypic changes. In this study, using DNA from the Southampton Women's Survey (SWS), we identified changes in DNA methylation levels at birth that associated with adiposity later in childhood within the promoter of ANRIL, a gene with strong disease associations that is involved in regulatiing cellular growth. This finding was replicated in several independent cohorts, and functionally validated in adipocyte cells.

## 1 Introduction

Fixed variations in a number of genes have been linked to obesity (Frayling et al., 2007, Loos et al., 2008, Thorleifsson et al., 2009), although to date single nucleotide polymorphisms and copy number variations explain only a fraction of the risk of obesity and metabolic disease in humans (Wellcome Trust Case Control, C, 2010). However, there is now substantial evidence from both human and animal studies that the quality of the early life environment before and after birth can affect susceptibility to metabolic disease in later life (Godfrey and Barker, 2001). In humans, famine exposure during pregnancy (Roseboom et al., 2006), maternal obesity (Catalano and Ehrenberg, 2006, Robinson et al., 2015), and gestational diabetes (Pettitt et al., 1991) are all associated with an increased risk of obesity in the offspring, while in animal studies variation in maternal diet has been linked to alterations in offspring metabolism and body composition (Bertram and Hanson, 2001, Poston, 2010).

Epigenetic regulation of gene function is one mechanism through which early life environmental factors could induce persistent phenotypic changes. For example, feeding rats a modest reduction in protein during pregnancy induced hypomethylation of the promoter regions of the nuclear receptors Glucocorticoid Receptor (*NR3C1*) and Peroxisome Proliferator activated receptor alpha (*PPARA*) in the liver of the offspring during adulthood, accompanied by increased *NR3C1* and *PPARA* gene expression, and alterations in the metabolic processes they control (Lillycrop et al., 2007). In humans, differences in the methylation of imprinted and non-imprinted genes related to cardio-metabolic phenotypes have been found in the peripheral blood leukocytes of adults whose mothers were exposed to famine during pregnancy (Heijmans et al., 2008). Consistent with the paradigm that developmentally induced epigenetic marks make a significant contribution to later phenotype, DNA methylation within the promoter of the retinoid X receptor alpha (*RXRA*) gene in umbilical cords predicted > 25% of the variation in later %fat mass in children (Godfrey et al., 2011), while PPAR-γ-co-activator-1α promoter methylation in blood at age 5–7 years predicted adiposity from 9 to 14 years (Clarke-Harris et al., 2014a). As the identification of such epigenetic changes may provide insights into the molecular mechanisms underlying the development of adiposity and allow the identification of individuals at increased risk of metabolic disease, we carried out a discovery genome-scale DNA methylation scan to identify differences in DNA methylation levels in umbilical cord from children from the Southampton Women's Survey (SWS) cohort associated with child's adiposity at age 6 years. We identified an association between the methylation level at birth of CpG loci within the promoter of ANRIL (antisense non-coding RNA in the *INK4* locus), a 3.8 kb non-coding RNA transcribed from the *CDKN2A* gene locus, and variation in total and %fat mass at 6 years. We also observed this association in cord tissue from ethnically diverse neonates from the Growing Up in Singapore with Healthy Outcomes (GUSTO) cohort (n = 187), in peripheral blood from adolescents (n = 812) in the Western Australian Pregnancy (RAINE) cohort, and in adipose tissue from adults (n = 81) from the UK BIOCLAIMS cohort. Finally, we showed that CpG methylation at this locus was associated with ANRIL expression *in vivo* and CpG mutagenesis *in vitro* inhibited ANRIL promoter activity. Furthermore, we identified an Estrogen Receptor-α (ERα) containing protein complex that binds to this region in a methylation specific manner and regulates transcription from this locus.

## 2 Methods

### 2.1 Southampton Women's Survey (SWS): Participants

The SWS is a prospective study that assessed the diet, body composition, physical activity and social circumstances of a large group of non-pregnant women aged 20–34 years in Southampton, UK. Comprehensive details have been published (Inskip et al., 2006). Women who subsequently became pregnant were followed up during pregnancy with serial measurements of fetal size and their offspring studied in infancy and childhood (Pike et al., 2010). There were 1981 women who became pregnant and delivered a live-born singleton infant before the end of 2003. Six infants died in the neonatal period and two had major congenital growth abnormalities, which left 1973 mother-offspring pairs. Triceps skinfold thickness at birth and age 1-year was measured using Holtain skinfold calipers (Holtain Ltd., Crymych, UK). A sub-set of children from the SWS study with dietary data in infancy was invited to take part in follow-up at ages 4 and 6-years to assess their body composition (Table S2). Adiposity measurements were made by dual-energy X-ray absorptiometry (DXA) (Hologic Discovery, paediatric scan mode, Hologic Inc., Bedford, MA) (Crozier et al., 2012). Follow-up of the children and sample collection/analysis was carried out under Institutional Review Board approval (Southampton and SW Hampshire Research Ethics Committee 06/Q1702/104) with written informed consent. Clinical investigations were conducted according to the principles expressed in the Declaration of Helsinki.

### 2.2 Growing Up in Singapore Towards healthy Outcome (GUSTO): Participants

The GUSTO mother-offspring study (Soh et al., 2013) collected biosamples and measured adiposity in babies born at the KK Women's and Children's Hospital (KKH) and the National University Hospital (NUH) in Singapore. Ethical approval for the study was granted by the centralized Institute Review Board (CIRB) and the Domain Specific Review Board (DSRB), the ethics boards of KKH and NUH, respectively (NCT01174875). 1247 women (response rate 61.3%) were recruited; participant characteristics are shown in Table S6. Infant weight was measured to the nearest gram on a calibrated scale (SECA 334, SECA Corp, Hamburg, Germany). Recumbent crown–heel length was measured using an infant mobile measuring mat (SECA 210, SECA Corp Hamburg, Germany). At age 18 months ponderal index (weight/length (Thorleifsson et al., 2009)) was derived as a measure of adiposity. Triceps and subscapular skinfold thickness were measured using Holtain skinfold calipers.

### 2.3 BIOCLAIMS Study

The BIOCLAIMS study was a randomised controlled clinical trial of 100 volunteers recruited in Southampton UK 2012–2013. Inclusion criteria were: men or women aged 18–65 years, BMI 18.5–25 (lean) or BMI 30–40 kg/m^2^ with waist circumference > 94 cm for men > 80 cm for women (obese), not eating more than one oily fish meal per week and being able to provide written informed consent. Exclusion criteria were: diagnosed diabetes, use of prescribed medicine to control inflammation, blood lipids or blood pressure, use of fish oil or other oil supplements, chronic gastrointestinal problems, pregnancy/planning pregnancy, or participation within another clinical trial. Adipose tissue biopsies were collected from 81 volunteers at baseline (lean n = 37, obese n = 44) (Table S12).

### 2.4 The West Australian Pregnancy Cohort (Raine Study): Participants

The Raine Study enrolled pregnant women ≤ 18th week of gestation (1989–1991) (N = 2900) through the antenatal clinic at King Edward Memorial Hospital and nearby private clinics in Perth, Western Australia. Detailed clinical assessments were performed at birth. Birth information (including birth weight and height) was obtained from midwife records. The children were followed up at multiple time points including at 17 years of age at which time physical assessments including weight, height, and skin fold assessments were performed as described previously (Huang et al., 2015). Socioeconomic status was assessed by maternal education. Maternal weight and height was measured by a trained midwife at 18 weeks gestation. Early pregnancy weight was obtained at recruitment around 18 weeks gestation. Gestational age was based on the date of the last menstrual period unless there was discordance with ultrasound biometry at the dating scan. The Human Ethics Committees (King Edward Memorial Hospital and/or Princess Margaret Hospital) approved all protocols (RA/4/1/6613). Informed, written consent to participate in the study was obtained from the mother of each child at enrolment and at each subsequent follow-up.

### 2.5 DNA Extraction

A 5–10 cm segment was cut from the mid portion of each cord, immediately following delivery, flushed with saline to remove fetal blood, flash-frozen in liquid nitrogen and stored at − 80 °C until required for DNA isolation. Genomic DNA was prepared from umbilical cord and cord blood by a standard high salt method, and from adipose tissue using the QIAamp DNA mini kit (Qiagen, Germany).

### 2.6 Whole Genome Methylation Analysis

**Fig. 1:**
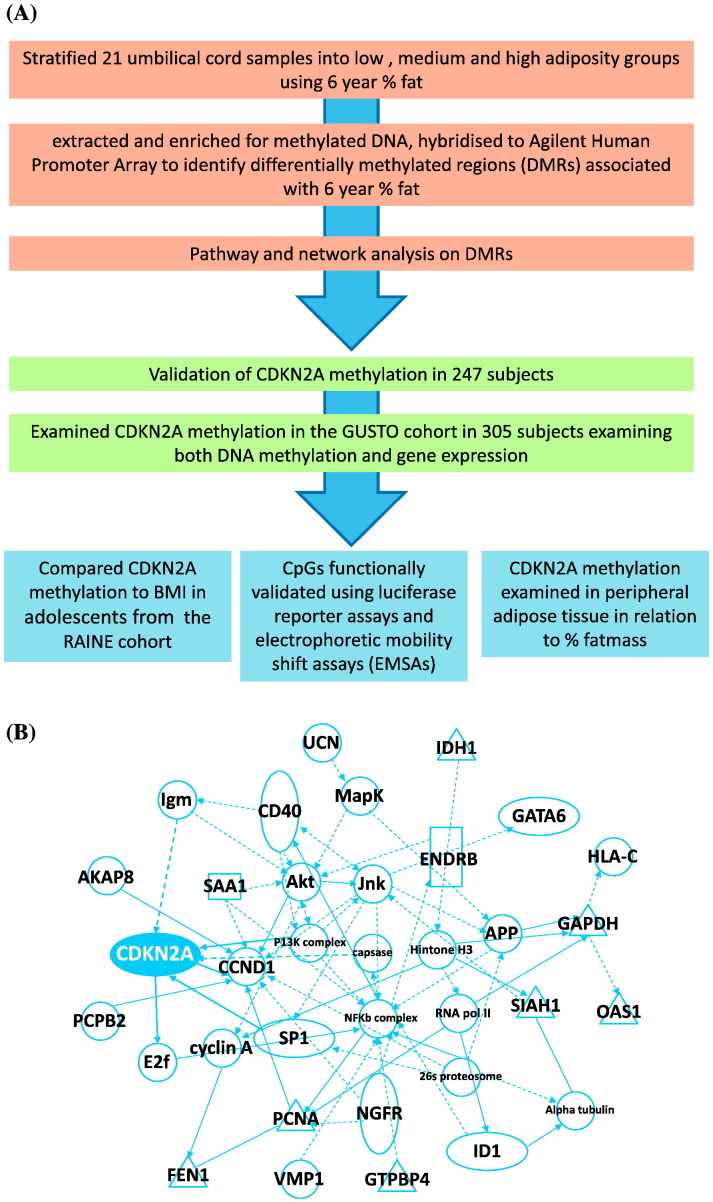
(A) An overview of the study. (B) The top network enriched amongst the DMRs was DNA replication, recombination and repair.

Genomic DNA from umbilical cord samples was obtained from 21 children from the SWS cohort, chosen to represent a range of %fat between the 5th and 95th percentiles for this population measured by DXA at age-6 years. DNA methylation levels were quantified using Agilent Human Promoter Whole-Genome ChIP-on-chip array (G4489A), and analysed using Bayesian Tool for Methylation Analysis (BATMAN) (Down et al., 2008) as previously described (Lillycrop et al., 2015). An overview of the study design in shown in Fig. 1A.

### 2.7 Statistical Tests to Identify Differentially Methylated Regions (DMRs)

Percentage methylation values at each 100 nt region were subjected to robust regression analysis percent fat to correct for heteroscedasticity (Barton et al., 2013). Fisher Exact tests were performed to identify larger chromosomal regions DMRs that tiled sequentially on the array. These regions were tested for significant enrichment of differential methylation amongst the 100 nt regions, within their span. This principal is similar to that of Jaffe et al. (2012) but is especially designed for the custom array design. The cut offs used to select DMRs were designed to be a stringent filter to prioritise genes for the pathway analysis.

### 2.8 Test for Robustness to Failed Probe Signals

It was observed that a portion of the probes included in the raw data had a log2 value approaching 0. These probes were assumed to be failed probes and were removed from the dataset and the modified dataset was subjected to the BATMAN algorithm. Any DMRs not robust between the two datasets were removed from consideration. A surrogate variable analysis (SVA) (Leek et al., 2012) similar to the “reference-free” analysis suggested by Houseman et al. (2014) was run to check for any confounding effects of differences in cellular heterogeneity.

### 2.9 IPA Analysis

Gene pathway analysis was carried out using Ingenuity Pathway Analysis software (Qiagen), with the design of the array set as the background in the pathway analysis. The gene list of identified DMRs from the BATMAN analysis was used to generate gene networks *via* a core analysis based upon the Ingenuity Pathways Knowledge Base.

### 2.10 Statistical Analysis

Statistical analysis on the SWS was carried out using Stata (Statacorp) versions 11.2 and 12.1. Histograms of all continuous variables were plotted to check for normality. The distributions of some measures of adiposity were skewed and therefore transformed using a log_e_ transformation. Conditional sex-specific abdominal circumference growth velocities (Z-scores) were calculated from antenatal ultrasound measurements as described previously (Pike et al., 2010). Velocities of prenatal and infant growth were calculated from change in size adjusted for gestation or age, as appropriate. DXA adiposity measurements were calculated without including the child's head to minimise the effect of head movement on the measurement. Regression models were built using CpG methylation as the predictor and child's adiposity measurement (at 4 or 6 years) as the outcome, and of conditional abdominal circumstance growth velocity from 34 weeks gestation to birth as the predictor and cord CpG methylation as the outcome. Models were adjusted for child's sex and age as appropriate for each time point. Offspring adiposity was measured as %fat and total fat in grams, converted to Z-scores to facilitate interpretation of the effect size, and methylation measurements as %; the regression coefficient can therefore be interpreted as the standard deviation change in %fat (or total fat) for each % change in methylation. Results are presented as regression coefficients (β), which represent the (mean) change in outcome for a one unit (%) change in methylation, and their associated p-values. Analyses for validation of the array results were carried out on the full range of data, without grouping.

As there was a strong correlation between methylation of the individual CpGs within ANRIL, and because the number of tests carried out was modest in comparison with genome-wide approaches, Bonferroni or Benjamini-Hochberg false discovery rate corrections for multiple testing would not be appropriate (Goeman and Solari, 2014). Instead a data reduction approach was employed by investigating the correlations between methylation at the 9 CpG sites, identifying those which cluster together statistically, and choosing the CpG with the highest median absolute deviation (MAD) score to represent that cluster. In both the SWS and GUSTO cohorts, the clustering of CpGs sites was similar with CpGs 1–2, 3 4–8 clustering. The exception was CpG9, which clustered with CpG 4–8 in the SWS cohort but separately in the GUSTO cohort. We therefore used a clustering which was appropriate across both cohorts, and grouped the 9 CpG sites into 4 clusters CpGs 1–2, 3, 4–8 and 9, represented by CpGs 2, 3, 7 and 9.

Linear regression was used to analyse associations between DNA methylation levels, gene expression and infant adiposity measures in the GUSTO cohort. DNA methylation and infant adiposity measures were adjusted for sex, ethnicity, maternal age, mode of delivery and cell type. Cell type was corrected for using cellular proportions estimated from a reference panel's cell-specific methylation dataset (Gutierrez-Arcelus et al., 2013, Houseman et al., 2012). Experimental batches were accounted for as a covariate. Similar linear regressions were done to assess the relationship between methylation and gene expression. All statistical analyses in the GUSTO cohort were performed in R (version 2.15.3).

Linear regression was used to analyse association between DNA methylation levels and BMI in the Raine cohort. The distribution of BMI in this cohort was positively skewed and therefore log-transformed. Multivariate linear regression was adjusted for age and sex. Differences in cellular heterogeneity in blood was assessed Cell type.

### 2.11 Pyrosequencing

The levels of DNA methylation at the *CDKN2A* DMR in DNA extracted from umbilical cord was measured by sodium bisulfite pyrosequencing as previously described (Lillycrop et al., 2015). Methylation ranges are shown in Table S13 and primers in Table S14.

### 2.12 Real-time PCR in the GUSTO Cohort

RNA was extracted from whole umbilical cords obtained in the GUSTO study, using the RNeasy Mini Kit (Qiagen) as per manufacturer's guidelines. cDNA was prepared using the Hi-Capacity cDNA Reverse Transcription Kit (Life Technologies), and qPCR reactions run in triplicate with Power SYBR (Life Technologies) on a ABI 7900HT as per manufacturer's guidelines. Primer sequences are shown in Table S14 Primers to detect circular transcripts are described in Burd et al. (2010). All Ct values were normalized to the geometric mean of TOP1 and PPIA reference amplicons, and thereafter referred to as dCt (delta-Ct) values. The dCt value was used in all subsequent regressions and statistical analyses, since assignment to a calibrator necessary for the exponential determination of fold change would be arbitrary and inappropriate for a longitudinal-type population with continuous characteristics.

### 2.13 Cloning of Promoter Regions

Two sets of PCR primers were used. The first set amplified the ANRIL promoter (− 926 to + 20 relative to ANRIL TSS); a *Hin*dIII restriction site was added to the forward primer, and an *Nco*I restriction site to the reverse primer for cloning into pGL Basic (Promega, UK) to create pGL − 951. The second PCR primer pair amplified a region containing the DMR, immediately adjacent to the already cloned ANRIL promoter region (− 1281 to − 925); a *Kpn*I restriction site was added to the forward primer, and a *Hin*dIII restriction site to the reverse primer for cloning into pGL − 951. The completed plasmid, pGL ANRIL, contained the full genomic sequence from − 1281 to + 20 of the ANRIL promoter with a *Hin*dIII site inserted after the DMR to allow its subsequent removal. All PCR amplification was carried out using Hot Star High Fidelity DNA polymerase (QIAGEN). Primers are listed in Table S14. The base pair sequence of the cloned region was confirmed by sequencing (GATC, Germany). The p14^ARF^ promoter region was produced by GeneArt (Applied Biosystems), and subsequently cloned into pGL3 Basic.

### 2.14 PCR Mutagenesis

Mutagenesis primers were designed using QuikChange Primer Design. PCR was carried out using the SequalPrep™ Long PCR Kit (Life Technologies A10498) following manufacturers guidelines. Mutagenesis was confirmed by sequencing (GATC, Germany).

### 2.15 Cell Culture and Transfection

SW872 cells were cultured in 24well plates (DMEM 4.5 g glucose, 10% FBS, 1%P/S) for 24 h prior to transfection. 500 ng plasmid DNA was transfected (six replicates per transfection). pGL CMV Renilla (Promega UK) was co-transfected as a control. Transfections were carried out using FuGENE HD (Switchgear Genomics USA) following manufacturers' guidelines. Transfected cells were cultured for 48 h prior to harvesting. Luciferase assays were carried out using the Dual-Luciferase® Reporter Assay System (Promega UK), on a VarioSkan Flash Luminometer (ThermoScientific). For estrogen treatment, cells were cultured in phenol-free media and charcol-stripped FBS for 48 h prior to addition of 10 nM E2 (Sigma E2758). Cells were cultured for 72 h then harvested for RNA.

### 2.16 Realtime PCR in SW872 Cells

RNA was extracted from whole umbilical cords obtained in the GUSTO study, using the RNeasy Mini Kit (Qiagen) as per manufacturer's guidelines. cDNA was prepared using Enhanced AMV-RT (Sigma) after DnaseI treatment (Sigma). Realtime PCR was carried out in gene expression master mix (Life technologies), and gene expression assayed for p14 and exons 5–6 for ANRIL (Life technologies p14 (Hs99999189, Hs04259476). Anril unspliced used custom primers (Table S14) amplifying a region of intron 1.

### 2.17 Electrophoretic Mobility Shift Assays

EMSA were carried out on nuclear extracts from SW872 cells as previously described (Clarke-Harris et al., 2014b). DNA oligonucleotides (Sigma Aldrich) are listed in Table S14.

## 3 Results

### 3.1 Differentially Methylated Regions (DMRs) at Birth Are Associated With Later Adiposity

To identify perinatal epigenetic biomarkers associated with later adiposity, we carried out a discovery scan of DNA methylation in the promoters of all refseq genes in umbilical cord DNA, comparing locus specific DNA methylation levels at birth with differences in percentage (%) fat mass in the children aged 6 years (Fig. 1A; Table S2). Methylated DNA was captured using the methyl-binding domain (MBD) of MBD2, before the enriched methylated DNA fraction and input DNA was hybridised to the Agilent Human Promoter Whole-Genome array (G4489A). DNA methylation levels were estimated, adjusting for CpG density, using the validated algorithm BATMAN (Down et al., 2008). This identified 93 differentially methylated regions (DMRs) associated with %fat mass in the children at 6 years of age measured by dual-energy X-ray absorptiometry (DXA) (Table S3). The top pathway enriched amongst the 93 DMRs was DNA replication and repair (p = 1 × 10^− 41^) (Fig. 1B, Table S4); 41 of the 93 DMRs were associated with this pathway. The DNA replication and repair pathway included DMRs linked with Cyclin dependent Kinase 2A inhibitor (*CDKN2A*), cyclin D (*CCND1*), flap structure-specific endonuclease 1 (*FEN1*), proliferating cell nuclear antigen (*PCNA*) and A-kinase anchor protein 8 (*AKAP8*) (Fig. 1B). Cell cycle regulators have been increasingly linked to the control of metabolism. The cell cycle inhibitors p16^INK4a^ (inhibitor of cyclin-dependent kinase 4) and P14^ARF^ (alternative reading frame relative to p16), encoded by the CDKN2A locus, have been shown to play a direct role in regulating adipocyte number, function and senescence (Abella et al., 2005). Furthermore multiple GWAS studies have identified the CDKN2A locus, particularly the region encoding the long non-coding RNA ANRIL which negatively regulates p16^INK4A^, as a hotspot for cardiovascular disease, type 2 diabetes, and frailty. However to date there have been no studies linking the differential methylation of *CDKN2A* or ANRIL with adiposity. We therefore further explored the association between the differentially methylated region within the CDKN2A locus at birth and child's %fat mass.

### 3.2 Lower *CDKN2A* Methylation is Associated With Increased Child Adiposity

**Fig. 2:**
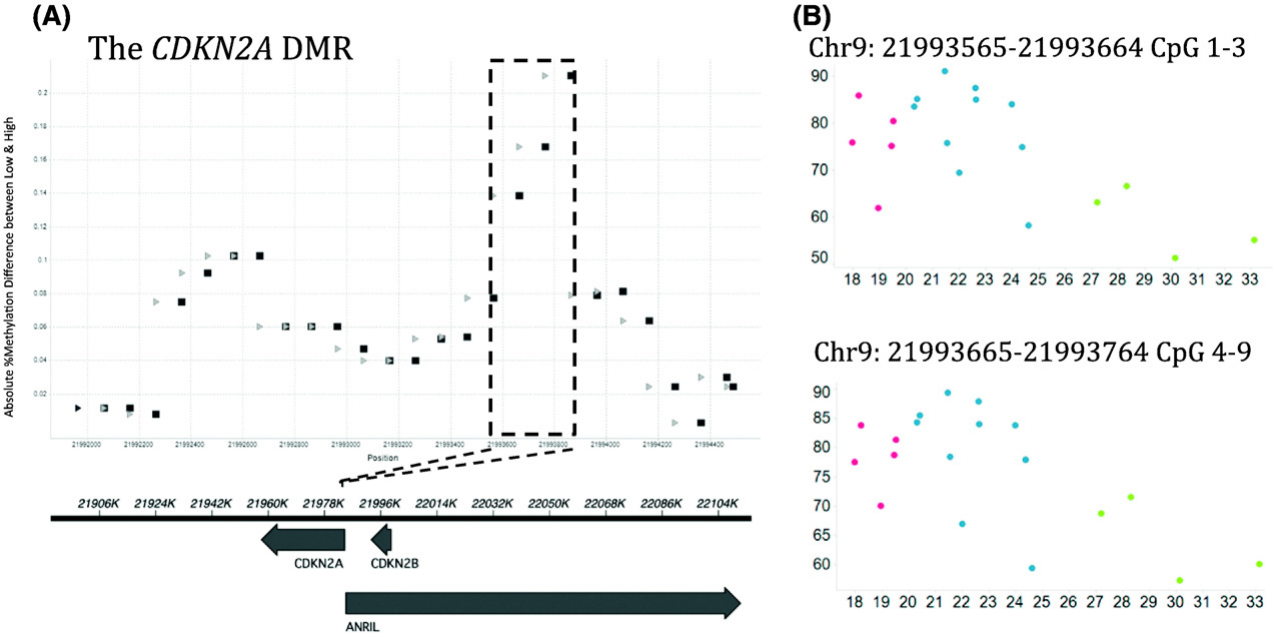
Association between *CDKN2A* DMR at birth and adiposity in childhood. (A) The DMR identified within *CDKN2A* (Chr9:21,993,565–21,993,764 (hg19). Percentage adiposity values for SWS subjects were divided into three groups from low (group 1) to high (group 3) %fat mass. The Y-axis shows absolute %methylation difference between groups 1 and 3. X-axis shows chromosomal coordinates (hg19). Light and dark grey circles represent start and end of each 100 nucleotide region returned from BATMAN, respectively. 100 nucleotide regions in the dotted box were found to have > 20% absolute methylation difference between groups 1 and 3 and these were the regions selected for pyrosequencing in the extended sample set. The lower panel shows the positions of gene transcripts. (B) The concordance of methylation values with percent body fat for *CDKN2A* using discovery data from the MBD whole genome promoter array. The X-axis shows percent body fat and y-axis shows % methylation as estimated by BATMAN. Percentage methylation values at each 100 nt region was subjected to robust regression analysis against % fat mass at 6 years of age. To illustrate the association found, sample data points are coloured by percent body fat group groups (red = low, lowest percent body fat; blue = medium, medium percent body fat; green = high, highest percent body fat.

**Fig. 3:**
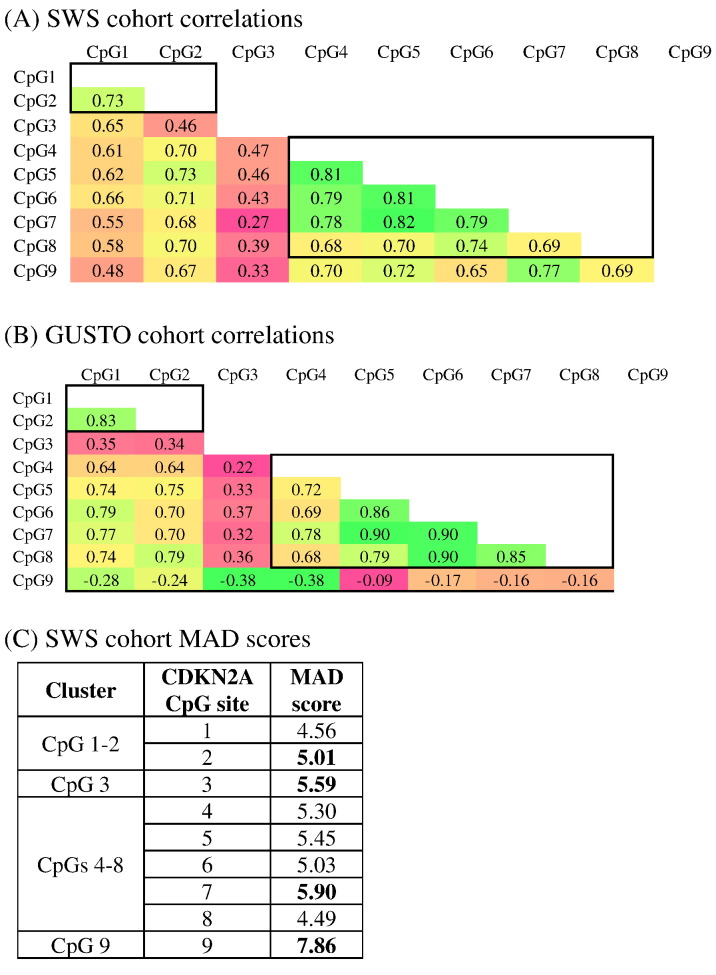
CpG Clustering. Spearman correlation of methylation levels at CpGs 1–9 within the ANRIL promoter. Four distinct clusters are defined: 1–2, 3, 4–8 and 9. (A) correlations in the SWS cohort. (B) correlations in the GUSTO cohort. (C) Median absolute deviation (MAD) scores within the 4 clusters in SWS Cohort.

Analysis of the promoter array revealed an association between lower methylation of the *CDKN2A* DMR at birth and higher %fat mass age 6-years (Fig. 2A–B). The association remained after array-wide adjustment for surrogate variables (Leek et al., 2012) (a procedure similar to that of Houseman et al., 2014), suggesting that cellular heterogeneity did not confound the association (Table S5). To validate the relationship between *CDKN2A* methylation and %fat mass age 6-years, sodium bisulfite pyrosequencing was carried out across the *CDKN2A* DMR in an independent group of infants from the SWS cohort (n = 247). The correlation between observed DNA methylation values at the 9 CpG dinucleotides were examined: two clusters of contiguous CpGs with highly correlated methylation levels (spearman rho > 0.68) could be determined within the *CDKN2A* DMR, CpGs 1–2 and CpGs 4–8 (Fig. 3A), Within these two clusters the CpGs with the highest median absolute deviation (MAD) score, CpG 2 and CpG 7, were selected as the representative CpG for those clusters in further analysis (Fig. 3C). As CpGs 3 and 9 showed some divergence from their neighbors they were tested individually.

Lower DNA methylation was associated with higher %fat mass age 6-years for CpG clusters 1–2 (p = 0.003), 3 (p = 0.007), and 4–8 (p = 0.042), but not CpG9 (p = 0.122). Lower DNA methylation was also associated with higher %fat mass age 4-years for cluster 1–2 (p = 0.007) and cluster 4–8 (p = 0.040) (Table 1). Data for the individual CpGs is also given in Table S6. For every 10% increase in perinatal *CDKN2A* CpG1 methylation there was a decrease in %fat mass of 0.20 SD (95% CI − 0.33, − 0.06) age 4 years and 0.18 SD (− 0.31, − 0.05) age 6 years, controlling for sex. Methylation of all four clusters was also associated with total fat mass ages 4 and 6-years (all p ≤ 0.03) (Table 1). We observed no associations between *CDKN2A* methylation and DXA %fat mass or total fat mass at birth (data not shown).

**Table 1:** Association between *CDKN2A* DMR methylation and percentage and total fat mass in the SWS cohort. Associations between % and total fat mass at birth, 4 years and 6 years for *CDKN2A*. Adjusted for sex, maternal age at birth and mode of delivery.

*CDKN2A*	4 year DXA: percentage fat			4 year. DXA: total fat			6 year. DXA: percentage fat			6 year DXA: total fat		
Cluster	n	β	p-Value	n	β	p-Value	n	β	p-Value	n	β	p-Value
1–2	215	− 0.09	**0.007**⁎⁎	215	− 0.005	**0.003**⁎⁎	204	− 0.004	**0.003**⁎⁎	205	− 0.005	**0.011**⁎
3	192	− 0.074	0.111	192	− 0.005	**0.024**⁎	184	− 0.005	**0.007**⁎⁎	184	− 0.007	**0.014**⁎
4–8	244	− 0.076	**0.04**⁎	244	− 0.004	**0.015**⁎	228	− 0.003	**0.042**⁎	229	− 0.006	**0.016**⁎
9	218	− 0.074	0.089	218	− 0.005	**0.011**⁎	203	− 0.003	0.122	204	− 0.007	**0.022**⁎

n = number of subjects, β = regression coefficient.

Results in bold have a significance ≥ 0.05

⁎ p = 0.05–0.01.

⁎⁎ p ≤ 0.01.

Genotyping was carried out for all known SNPs within the DMR region to investigate whether genetic variation explained the observed association between methylation levels and adiposity. Of the five SNPs analysed, three SNPs (rs192633385, rs190416574, rs187674321) were monomorphic in this population, while SNPs rs149570278 and rs111690342 were heterozygous with frequencies of 1.4% and 1.7% respectively; analysis showed no impact on the association between methylation and adiposity (data not shown).

### 3.3 *CDKN2A* Methylation is Associated With Triceps Skinfold Thickness in Early Infancy

*CDKN2A* methylation was associated with DXA measures of adiposity at 4 and 6 years of age, but not at birth; to examine this temporal difference in associations, we related perinatal *CDKN2A* methylation to triceps skinfold thickness, measured immediately after birth and at age 1-year. There was no association between DMR methylation and triceps skinfold thickness at birth, but an inverse association between methylation of CpG clusters 1–2, 3 and 4–8 with triceps skinfold thickness at age 1-year (p = 0.039, p = 0.068 and p = 0.014 respectively) (Table 2A). As one pathway to obesity has been suggested to involve fetal undernutrition followed by rapid postnatal weight gain, we used longitudinal measurements of fetal growth from the SWS cohort to derive conditional abdominal growth velocity in late gestation and related this to cord *CDKN2A* methylation. Here we found that fetal growth faltering between week 34 of gestation and birth was associated with lower *CDKN2A* methylation (Fig. S1, Table S1).

**Table 2:** Association between *CDKN2A* methylation at birth and triceps skinfold thickness in childhood. Methylation levels of the 9 CpG dinucleotides within the *CDKN2A* DMR were related to triceps skinfold thickness in (A) the SWS cohort at birth and 1-year (linear regression, adjusted for sex), and (B) the GUSTO cohort at day 7 and 18 months (adjusted for sex, ethnicity and cell type); n = number of subjects, b = regression coefficient.

(A)				
SWS cohort		Triceps skinfold thickness		
Cluster	Timepoint	n	β	p-Value
1–2	Birth	258	0.001	0.406
	1 year	258	− 0.032	**0.039**⁎
3	Birth	233	0.001	0.611
	1 year	235	− 0.037	0.068
4–8	Birth	295	0.002	0.119
	1 year	295	− 0.04	**0.014**⁎
9	Birth	267	0.001	0.436
	1 year	266	− 0.028	0.157

(B)				

GUSTO cohort		Triceps skinfold thickness		
Cluster	Timepoint	n	β	p-value

1–2	day 7	242	− 0.592	0.84
	18 month	215	3.872	0.29
3	day 7	217	− 9.557	0.004⁎⁎
	18 month	195	− 2.452	0.56
4–8	day 7	282	− 1.378	0.38
	18 month	253	− 0.021	0.99
9	day 7	204	− 3.718	0.17
	18 month	175	2.094	0.54

Results in bold have a significance ≥ 0.05

⁎ p = 0.05–0.01.

⁎⁎ p ≤ 0.01.

### 3.4 *CDKN2A* Methylation in Singaporean Infants is Associated With Adiposity

We examined DNA methylation of the 4 CpG clusters in umbilical cord DNA from the GUSTO cohort (n = 305), an independent Singaporean mother-offspring cohort where triceps and subscapular skinfold thickness had been measured at age 7-days and ponderal index age 18-months (Table 2B and Table S8). Analysis of GUSTO methylation levels at the nine CpGs showed a similar pattern of relatedness amongst CpGs (Fig. 3B). There was an even more divergent methylation profile at CpG9, suggesting the four test variables consisting of the two CpGs representative of larger contiguous correlated clusters and the two individual CpGs were appropriate across cohorts (Fig. S1C). Lower methylation of *CDKN2A* CpG cluster 3 (p = 0.004), was associated with greater triceps skinfold thickness at age 7-days (Table 2B), while associations or trends were also seen between lower methylation of CpG 3 (p = 0.004), and 9 (p = 0.08) and higher subscapular skinfold thickness at age 7 days, and between lower methylation of CpG 9 (p = 0.005) and greater ponderal index at age 18 months (Table S8), after adjustment for sex, maternal age, ethnicity, mode of delivery and cell type.

### 3.5 *CDKN2A* Methylation is Associated With the Expression of p16^INK4a^, P14^ARF^ and ANRIL Expression in Umbilical Cord

As the *CDKN2A* gene locus encodes for the cell cycle inhibitors p14^ARF^ and p16^INK4a^, as well as ANRIL, a long non-coding RNA, we investigated whether the methylation status of the CpG loci within the *CDKN2A* DMR was associated with the expression of transcripts generated from this gene locus in umbilical cord samples from the GUSTO cohort, for which both DNA and RNA was available. Expression of the unspliced form of the long non-coding RNA ANRIL was positively associated with the methylation of CpG cluster 9 (p = 0.02), while the spliced linear form of ANRIL was positively associated with the methylation of CpG cluster 4–8 (p = 0.02) (Table 3). There was no association between expression of the circular form of ANRIL and methylation of the DMR CpG clusters. In contrast, there was an inverse association between methylation of CpG cluster 9 and both p16^INK4a^ and p14^ARF^ expression (both p = 0.01).

**Table 3:** Gene expression correlates with *CDKN2A* DMR methylation in the GUSTO cohort. Methylation levels of the 9 *CDKN2A* DMR CpGs were correlated with expression levels (dCt values from RT-PCR) of the different ANRIL transcript variants, p14^ARF^ and p16^INK4a^ using linear regression, adjusted for gender and ethnicity; n = no. of subjects.

CpG cluster	ANRIL (linear)		ANRIL (circular)		ANRIL (unspliced)		p14ARF		p16INK4a	
	n	p-Value	n	p-Value	n	p-Value	n	p-Value	n	p-Value
1–2	174	0.06	116	0.539	174	0.70	174	0.98	137	0.07
3	167	0.24	110	0.940	167	0.27	167	0.78	130	0.06
4–8	180	**0.02**	118	0.051	180	0.06	180	0.91	142	0.95
9	106	0.16	81	0.111	106	**0.02**	107	**0.05**	91	**0.01**

Results in bold have a significance ≥ 0.05

As the methylation of CpGs within the DMR of *CDKN2A* DMR was associated with expression of ANRIL, p16^INK4a^ and p14^ARF^ transcripts, we also examined whether the expression of these transcripts was associated with infant adiposity (Table S9). The expression of the circular and unspliced forms of ANRIL were positively associated with triceps (p = 0.02 and p = 0.01) and subscapular skinfold (p = 0.05 and p = 0.01) thickness at day 7 (D7) in GUSTO infants. In contrast, there was a negative association between p14^ARF^ expression and day 7 ponderal index measurements (p = 0.04), while expression of p16^INK4^ was positively associated with both triceps (p = 0.04) and subscapular (p = 0.02) skinfold thickness at 18 months.

### 3.6 *CDKN2A* Methylation in Whole Blood DNA is Associated With BMI

Having observed an association between *CDKN2A* DMR methylation at birth with measures of adiposity in infancy and childhood, we next investigated whether *CDKN2A* methylation was associated with adiposity at an older age. DMR CpG methylation was measured in peripheral blood samples from adolescents aged 17-years from the Western Australian RAINE mother-offspring cohort (n = 814), examining the correlation between *CDKN2A* methylation in peripheral blood and adiposity. Analysis of the 9 CpGs showed CpG clusters with highly correlated methylation levels (Fig. S1D) similar to those observed in SWS and GUSTO. Adjusting for age and sex, lower methylation of CpG clusters 4–8 and 9 was associated with higher BMI (p = 0.008 and p = 0.006), with a trend observed between CpG3 and BMI (p = 0.06) (Table 4). Blood cell counts were measured in these samples but due to an observed strong collinearity (Table S11A and B) between blood cell counts and BMI (Variance Inflation Factors were extremely high (25 to 113.7) (Table S11C) increasing the variance of the regression coefficients and making them unstable), it was not therefore possible to adjust the association between methylation and BMI for cell type.

**Table 4:** Association between *CDKN2A* DMR methylation is with anthropometry at 17 years in the RAINE cohort. Methylation levels of the 9 CpG dinucleotides within the *CDKN2A* DMR were associated with BMI (linear regression adjusted for sex, age of child, and estimated cell counts - top two principal components); n = number of subjects, b = regression coefficient. BMI was natural log transformed.

		BMI (age and sex adjusted only)	
CpG cluster	n	β	p value
CpG 1–2	758	0.004	0.98
CpG3	723	− 0.004	0.060
CpG 4–8	740	− 0.004	**0.008**
CpG9	760	− 0.004	**0.006**

Results in bold have a significance ≥ 0.05

### 3.7 *CDKN2A* Methylation is Reduced in Adipose Tissue of Obese Adults

**Fig. 4:**
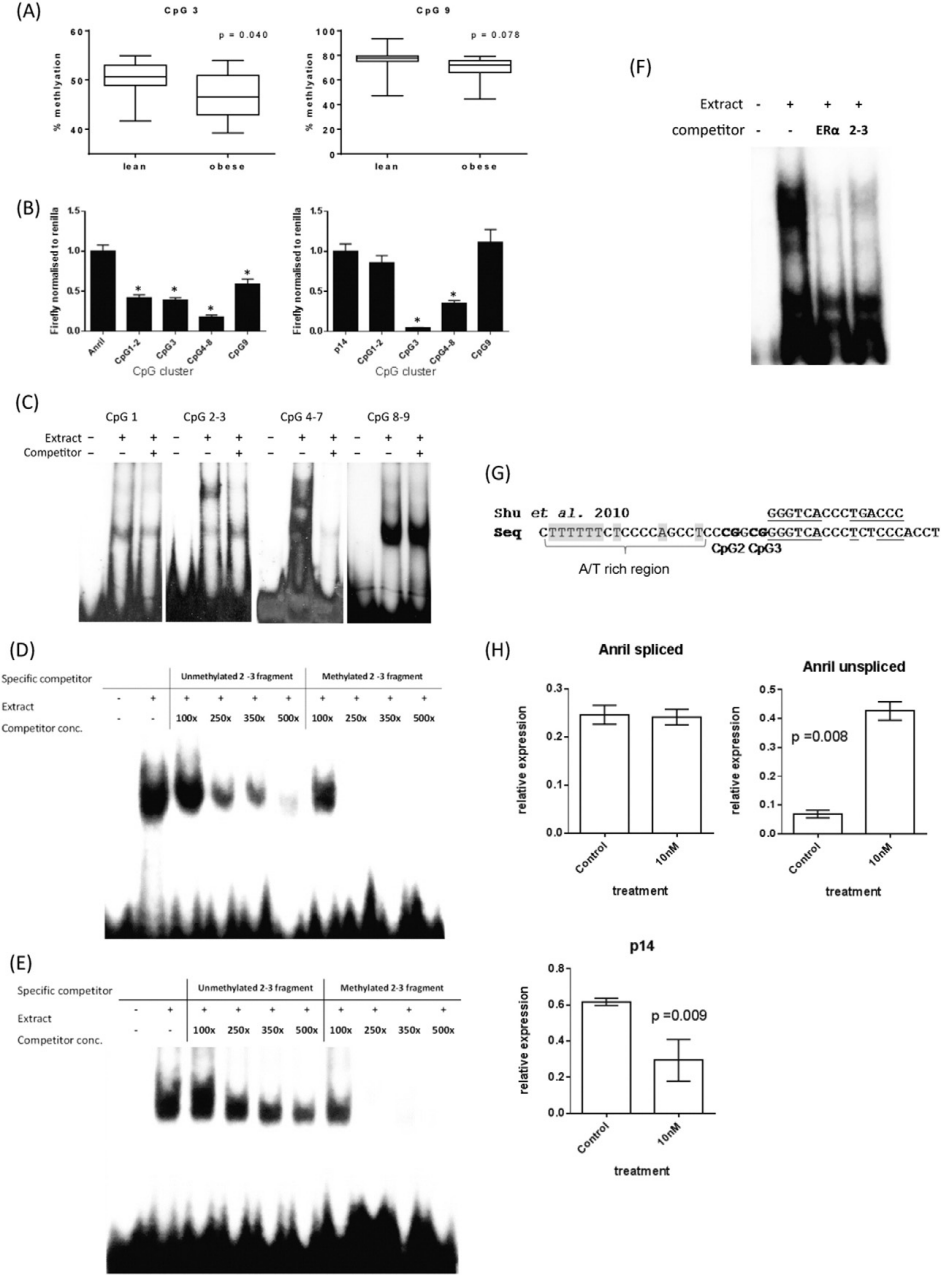
The role of CpGs in the *CDKN2A* DMR (A) Adipose methylation levels, lean *vs* obese groups, controlling for sex and age. (B) The ANRIL promoter and 5′ portion of the p14ARF gene were fused to the luciferase gene, and site directed mutagenesis of CpG sites performed. Constructs were transfected into the liposarcoma cell line SW-872 and luciferase activity measured. 6 technical replicates were undertaken per construct, per experiment and results combined from two independent experiments *p < 0.05, **p < 0.01, ***p < 0.005, ****p < 0.001. (C) EMSAs using SW-872 nuclear extract to examine differential binding within the DMR. (D) Varying levels of non-radiolabelled competitor was used to compete out binding to the radiolabelled probe covering CpG sites 2–3. (E) Unmethylated and methylated competitors were used to compare the effect of methyl groups on binding affinity (methylated at CpG2). (F) Binding of the 2–3 competitor compared using a radiolabelled probe methylated at CpG2. (F) CpG2–3 and ERα cold competitors were used to compete out binding of radiolabelled ERα probe. (G) ERE identified in the CpG2–3 region of the *CDKN2A* DMR, figure shows consensus ERE derived from Shu et al. top 643 confirmed EREs. (H) Expression of ANRIL and p14 in SW872 cells treated with 10 nM β-Estradiol for 72 h. ANRIL spliced primers detect Exons 5–6.

Having found an association between *CDKN2A* methylation in DNA from peripheral tissues and measures of adiposity, we next investigated whether these same CpG loci were differentially methylated in subcutaneous adipose tissue of obese compared to lean adults from the BIOCLAIMS cohort (http://bioclaims.uib.eu) (Table S11). %fat mass was used to characterize subjects as lean (12.2–20.9% for males and 17.3–31.5% for females) or obese (26.8–38% for males, 36.8–52% for females). Controlling for sex and age, methylation of CpG 3 was lower in adipose tissue from obese individuals compared to lean individuals (p = 0.04), with a trend also being observed for the methylation CpG9 (p = 0.078) (Fig. 4A). CpG Clustering based upon correlation of methylation was similar to previously observed (Fig. S1E).

### 3.8 CpG Sites Within *CDKN2A* Regulate ANRIL and p14^ARF^ Promoter Activity

As the identified DMR is in close proximity to the transcriptional start site of ANRIL and within the first exon of p14^ARF^, we investigated whether the DMR CpG sites may play a role in directly regulating the expression of ANRIL and/or p14^ARF^. The promoter region of ANRIL (− 1281 bp to + 20 bp relative to TSS) and the 5′ portion of the p14^ARF^ gene (− 500 bp to + 1125 bp relative to TSS) were fused to the reporter gene Luciferase in the vector pGL3basic, and CpG sites 2, 3, 7 and 9 individually mutated (CpG > TpG) then transfected into the liposarcoma cell line SW-872. Mutation of each of the 4 CpG sites decreased ANRIL promoter activity (all p ≤ 0.008) (Fig. 4B). In contrast, only mutation of CpGs 3 and 7 within the context of p14^ARF^ caused a decrease in expression, while alteration of CpGs 2 and 9 had no impact on expression.

### 3.9 DNA Methylation Alters Protein Complex Binding Within the DMR

Having shown that CpG loci associated with adiposity can influence the level of ANRIL and p14^ARF^ promoter activity, we used electrophoretic mobility shift assays (EMSAs) to determine whether methylation affected transcription factor binding to this region. The region containing the CpGs of interest was sub-divided into four regions based upon CpG chromosomal position. Incubation of nuclear extracts from SW-872 liposarcoma cells with oligonucleotides covering CpG1, CpGs 2–3, CpGs 4–7 and CpGs 8–9 revealed protein binding to each of the four regions, with binding to the oligonucleotides CpGs 2–3 and CpGs 4–7 competed out by an excess of unlabeled specific competitor (Fig. 4C). To determine whether methylation at the CpG 2–3 site affected binding to this sequence, an oligonucleotide containing CpGs 2–3 was incubated with nuclear extracts from liposarcoma cells with 50, 100 and 500-fold excess of either the unmethylated or methylated specific competitor containing a methylated cytosine at CpG2. While binding to the probe was markedly reduced in the presence of a 500-fold excess of the unmethylated specific competitor, only a 50-fold excess of the methylated specific competitor was required to effectively compete out binding (Fig. 4D), suggesting that methylation at CpG2 enhances DNA-protein binding. This marked increase in affinity of the protein complex for the methylated sequence was also observed when radiolabelled methylated probe was incubated with increasing concentrations of the unmethylated or methylated competitors (Fig. 4E).

### 3.10 The CpG 2–3 Region Contains an Estrogen Response Element

To identify the complex binding across CpGs 2–3, multiplexed competitor EMSAs were carried out. Multiplexed oligonucleotide DNA consensus sequences for eighty common transcription factors (Smith and Humphries, 2009) were used as competitors against the CpG 2–3 probe to identify potential transcription factors that may bind within the region. The protein complex bound across CpGs 2–3 was effectively competed out by the ERα consensus binding sequence. Consistent with this, addition of the CpG 2–3 unlabelled competitor successfully competed out binding to radiolabelled ERα probe (Fig. 4F). DNA sequence analysis of this region revealed the core consensus sequence (GGGTCACCCTCTCCC) for an ERα Estrogen Response Element (ERE) immediately downstream of the CpG 2–3 region matching known ERE conformations (Turner and Kinsella, 2010, Shu et al., 2010), and an upstream AT rich sequence, known to be important for ERα binding (Anolik et al., 1995) (Fig. 4G). To examine if estrogen can effect ANRIL expression, SW872 cells were treated with 10 nM β-Estradiol (E2) for 72 h. E2 treatment induced a 4-fold increase in expression of unspliced ANRIL (p = 0.008). Estrogen treatment decreased p14^ARF^ expression by 2 fold (Fig. 4H).

## 4 Discussion

Differential methylation of specific CpG loci within the promoter region of ANRIL at the *CDKN2A* gene locus was predictive of measures of adiposity in four independent cohorts. In the UK SWS cohort (n = 247), umbilical cord *CDKN2A* methylation was negatively associated with %fat mass at ages 4 and 6-years, in the Singapore GUSTO cohort (n = 305), it was negatively associated with skinfold thickness or ponderal index at ages 7 days and 18 months, in the Australian RAINE cohort (n = 814), whole blood *CDKN2A* methylation was negatively associated with concurrent BMI in 17 year olds and in the UK BIOCLAIMS cohort (n = 81) obese individuals had lower adipose tissue *CDKN2A* methylation compared to lean individuals. Although the negative association between *CDKN2A* methylation and adiposity measures was observed across the 4 cohorts, in 3 tissues and 3 age groups, there were differences in the nature of the relations. In the SWS cohort, lower umbilical cord *CDKN2A* DMR methylation predicted increased adiposity at ages 1, 4 and 6 years but not at birth, suggesting that *CDKN2A* methylation may be linked to the gain of fat mass postnatally. Two developmental pathways to obesity have been suggested, one associated with fetal undernutrition, followed by rapid postnatal weight gain, while the second pathway is linked to fetal overnutrition and greater fat mass at birth (Crozier et al., 2012, Crozier et al., 2010). Interestingly, fetal growth faltering in late gestation was associated with lower *CDKN2A* methylation at birth, suggesting that *CDKN2A* methylation may be a marker for the former pathway. In the GUSTO cohort, there were associations between the methylation of *CDKN2A* with measures of adiposity from 7 days after birth. The timing of the associations between the cohorts may reflect differences in fat deposition or rate of body fat accumulation between birth and early infancy, potentially due to genetic and epigenetic ancestral differences between the different cohorts that can affect timing of associations. The associations between the methylation of the individual CpGs within the *CDKN2A* DMR and adiposity were also not identical across the cohorts. The methylation status of CpG sites is often closely aligned to that of their neighbors, however developmentally induced changes are often CpG site specific (Lillycrop et al., 2008). In the DMR examined, the CpGs were divided into distinct groupings based upon strong inter-correlation, but these groupings had distinct differences between them, suggestive of tissue or population differences. This is re-iterated by the EMSAs, which showed that different protein complexes bind across the CpG sites within the DMR, suggesting they are likely to be differentially regulated. This is also supported by the *in vitro* studies, which showed that mutagenesis of the individual CpG sites had differential quantitative effects on expression.

An inverse association between *CDKN2A* methylation in umbilical cord and measures of adiposity was observed in the SWS and GUSTO cohorts. Surrogate variable analysis to control for cellular heterogeneity in cord tissue had little effect on our findings suggesting that, in cord, differences in *CDKN2A* methylation are not dependent upon variation in cellular heterogeneity. An inverse association between *CDKN2A* methylation and obesity was found in adipose tissue from adults, suggesting that differential *CDKN2A* methylation may be a robust marker of adiposity across cord and adipose tissue. The negative association between *CDKN2A* methylation and BMI was again observed in peripheral blood samples from the RAINE cohort. Blood is a heterogeneous material with dynamic cellular proportions and DNA methylation is differential across cell types, making it challenging to resolve the causal relationships between methylation of whole tissue, cellular mix and phenotype. Cellular proportions were measured in the RAINE cohort, however in this (Table S11A) and other studies, cellular proportions strongly associate with BMI. As would be expected methylation levels also differ across cell types (Table S11B). Multicollinearity was seen between all cell types and log BMI for all CpG clusters within the *CDKN2A* DMR. Therefore adjustment of the association between methylation and BMI for cellular proportions would violate the assumption of non-collinearity implicit in regression analysis. Consequently, we were unable to assess the dependence on cellular heterogeneity of the association of *CDKN2A* methylation in blood and BMI, and therefore cannot exclude differences in cellular composition as the explanation for the association between *CDKN2A* methylation in peripheral blood and adiposity in late adolescence. However; the existence of this association in very different tissues, which are (in the case of adipose) more homogenous in their cellular composition, and the fact that in umbilical cord it survived adjustment for cellular heterogeneity, suggests that the relationship is not completely explained by the confounding influence of cell type.

The *CDKN2A* gene locus encodes for p14^ARF^, p16^INK4a^, and the long non-coding RNA *ANRIL,* which negatively regulates p16^INK4a^. p16^INK4a^ inhibits CDK4 and CDK6 activity, which regulate the RB-E2F axis and cell cycle progression (Nilsson and Landberg, 2006, Jin et al., 1995, Jacobs et al., 1999, Abella et al., 2005), while p14^ARF^ also induces cell cycle arrest through its interaction with MDM2, resulting in increased p53 levels that trigger cell cycle arrest at both G1 and G2/M phases (Pomerantz et al., 1998, Zhang et al., 1998). Both p16^INK4a^ and p14^ARF^ also play roles in driving cellular senescence and ageing (Kim and Sharpless, 2006, Matheu et al., 2009). There is also evidence that the p16^INK4a^-cdk4-pRB axis plays a direct role in metabolic regulation and adipocyte differentiation (Aguilar and Fajas, 2010). Recent studies of genetically engineered mice deficient in E2F1, cdk4, and pRB, have shown that they contribute to lipid synthesis, glucose production, insulin secretion, and glycolytic metabolism. For example, adipose tissue-specific inactivation of the retinoblastoma protein induced increased mitochondrial activity, resulting in increased energy expenditure, which protected from diet-induced obesity (Dali-Youcef et al., 2007). In addition, inactivation of cdk4, which controls the clonal expansion phase of adipogenesis and phosphorylates PPARγ, blocked adipocyte differentiation, while a Cdk4 mutant, which cannot be inhibited by p16^INK4a^, increased the adipogenic potential of 3T3-L1 cells (Abella et al., 2005).

Data from the BLUEPRINT epigenome project suggests that the *CDKN2A* DMR overlaps with regulatory sequences. In monocytes, the DMR lies within a DNase hypersensitive peak and is associated with H3K27me3, H3K4me1 and H3K4me3 marks. The existence of both repressive and activating histone marks suggests that a bivalent domain is located across the region, while in B cells and HUVECs, there is a peak of H3Kme1, a mark often enriched at enhancers or regulatory sequences. Consistent with sequences within the DMR having a functional role in the regulation of *CDKN2A* directed transcription, in the GUSTO cohort, where both DNA and RNA were available from the cord tissue, there were associations between the methylation of CpG cluster 4–8 and CpG 9 and the expression of the *CDKN2A* generated transcripts, suggesting that differential CpG methylation within the promoter of ANRIL may have downstream effects on p16^INK4a^ and p14^ARF^ expression. Here, lower CpG methylation was associated with a decrease in ANRIL expression and an increase in p16^INK4A^ and P14^ARF^ expression. Increases in p16^INK4a^ and p14^ARF^ expression would be expected to lead to a decrease in cell proliferation and increased cellular senescence; if re-capitulated in adipocytes, this could promote both a decrease in adipocyte number and impaired adipocyte function, since p16^INK4a^ regulates cdk4 activity, which in turn phosphorylates and activates the adipogenic transcription factor PPARγ. Interestingly the region encoding ANRIL has been identified by GWAS as a hot spot for genetic variation associated with a number of ageing associated diseases, such as CVD and T2D. Sequence alterations within ANRIL may similarly affect p16^INK4a^ expression with downstream effects on cellular senescence, impairing the regenerative potential of cells, and increasing susceptibility to ageing associated diseases. Whether differential methylation of this promoter region of ANRIL or other CpGs sites further upstream also associated with an altered susceptibility to other ageing associated diseases is, as yet, unclear. Thus, these findings suggest that both genetic and epigenetic alterations within ANRIL may have important consequences for future disease risk.

In liposarcoma cells, we found that site directed mutagenesis of CpG sites affected ANRIL and p14^ARF^ promoter activity respectively, and although mutation of a CpG site does not infer that methylation of the CpG would have the same effect, it does clearly suggest that these CpG sequences are important determinants regulating ANRIL and p14^ARF^ expression. Binding of specific protein complexes to sequences within the DMR was also observed in liposarcoma cells, with methylation of CpG 2 increasing protein binding. DNA methylation has generally been associated with reduced transcription factor binding (Tate and Bird, 1993), but there is now growing evidence that DNA methylation can also enhance transcription factor binding (Clarke-Harris et al., 2014b). Interestingly, multiplexed competitor EMSAs identified ERα binding across CpGs 2–3. ERα plays an important role in maintenance of metabolic homeostasis and insulin sensitivity, with ERα perturbation linked to the metabolic syndrome (Hevener et al., 2015). ERα is highly expressed in adipose tissue (Mizutani et al., 1994), and a reduction in expression is linked to adipocyte hyperplasia and hypertrophy, insulin resistance and glucose intolerance in both sexes (Mauvais-Jarvis et al., 2013). We found that treating liposarcoma cells with estrogen stimulated expression of ANRIL, and reduced p14^ARF^ expression. While it remains unclear if ANRIL or p14^ARF^ are direct targets of ERα *in vivo* or part of the pathway by which ERα influences adipocyte function, our experiments do suggest that lower DMR methylation would decrease ERα binding and increase adiposity, consistent with studies showing increased adiposity in ERα knock out mice and that ANRIL may be an important component of this pathway.

Limitations of this study relate to the lack of cord, blood and adipose tissue from the same individuals, so we cannot extrapolate how *CDKN2A* methylation in cord tissue may relate to the methylation of *CDKN2A* in adipose or blood in the same individual, and/or whether such epigenetic alterations are causally involved in the development of fat mass. *CDKN2A* methylation was also measured in the 4 cohorts at different ages making exact comparisons between the cohorts more difficult, but the fact that a consistent negative association between *CDKN2A* methylation and measures of adiposity was seen across tissues types and ages suggest that differential methylation of *CDKN2A* may be a robust marker of adiposity.

Interactions of genotype and *in utero* environment best explain the majority of inter-individual differences in umbilical cord DNA methylation (Teh et al., 2014). Therefore *CDKN2A* methylation levels may be specified by an interaction of infant genotype with *in utero* environment or genotype alone (known as a methylation quantitative trait loci (methQTL). MethQTLs are overwhelmingly found *in cis* and the peak enrichment for SNP to CpG distance is within 45 bp (Gibbs et al., 2010). We excluded all known SNPs within the *CDKN2A* DMR (encompassing > 45 nt) by direct sequencing; without genome-wide sequencing it is not possible to exclude effects or epistatic interactions of distant SNPs, but the inter-individual variations in *CDKN2A* methylation are most likely to be a product of differential *in utero* environments, perhaps in interaction with individual genotype.

Genome wide association studies (GWAS) have shown that SNPs in the *CDKN2A* locus (typified by rs10757274) and encompassed by ANRIL, but 94 KB downstream of the DMR discussed in this paper, were associated with increased susceptibility to frailty, coronary artery disease, myocardial infarction, type 2 diabetes and late onset Alzheimer disease (Melzer et al., 2007, Cunnington et al., 2010, Grarup et al., 2007, Zhuang et al., 2012, Zeggini et al., 2007, Scott et al., 2007, Broadbent et al., 2008, Congrains et al., 2012). Interestingly the genetic associations seem independent of obesity (Broadbent et al., 2008). Disease associated genotype is also associated with ANRIL expression levels, and ANRIL expression also differs relative to disease (Zhao et al., 2015, Shanker et al., 2014, Bochenek et al., 2013). Previously Zhuang et al. (2012) found that methylation in blood at a locus overlapping with the DMR discussed here, was higher in coronary artery disease compared to controls but methylation was not associated with genotype at rs10757274 (Zhuang et al., 2012). This leads us to speculate that both rs10757274 risk genotype and prenatal environment mediated by differential methylation independently affect ANRIL expression to mediate disease risk.

The finding of a consistent association between *CDKN2A* methylation at birth and later adiposity in Caucasian and Asian cohorts at loci relevant to gene function provides substantial support for a role for epigenetics in mediating the long-term consequences of the early life environment on health, and suggests that altered methylation of CpG loci within the *CDKN2A* locus provide a robust prognostic marker of adiposity trajectory. Moreover these findings suggest a role for the long non-coding RNA ANRIL in the developmental origins of obesity, and identify estrogen as a novel regulator of ANRIL expression.

## Funding Sources

This work was supported by funding from the Medical Research Council (MC_UU_12011/4, MC_U147585827 and MC_ST_U12055) British Heart Foundation (RG/15/17/3174 and RG/07/009), Nestec (BIDG/2013/00456), NIHR Musculoskeletal Biomedical Research Unit, University of Oxford, NIHR Southampton Biomedical Research Centre, University of Southampton and University Hospital Southampton NHS Foundation Trust, the Singapore National Research Foundation under its Translational and Clinical Research (TCR) Flagship Programme administered by the Singapore Ministry of Health's National Medical Research Council (NMRC) (NMRC/TCN/012-NUHS/2014), Singapore- NMRC/TCR/004-NUS/2008; NMRC/TCR/012-NUHS/2014. The BIOCLAIMS study was supported by the European Commission Seventh Framework Programme (Grant agreement no. 244995). Additional funding was provided by the Singapore Institute for Clinical Sciences, Agency for Science Technology and Research (A*STAR), Singapore. KMG is supported by the European Union Seventh Framework Programme (FP7/2007-2013), project EarlyNutrition under grant agreement no. 289346. The RAINE study was supported by The Australian National Health and Medical Research Council (NHMRC) (1059711).

## Conflicts of Interest

The following authors have no financial interest related to this work: K.L., R.M., C.C., A.T., R.C.-H., S.B., P.C., E.G., E.C., P.T., B.S., S.L., Y-C.C., X.L., Y.W., G.B., C.C., H.I., N.K., J.H., C.E.C., C.P.C., Y.P., Y.L., Y.C., P.M., L.B., R-C.H., P.G., N.H., M.H., J.D., P.D.G. Both P.C.C. and K.M.G. have received travel reimbursement for speaking at conferences sponsored by companies selling nutritional and pharmaceutical products. This work was supported in part by a grant from Nestec.

## Authors' Contributions

R.M., R.C.-H., P.C., E.G., E.C. and C.Y.C. carried out the molecular epigenetic studies. R.M., B.S., S.L., Y-C.C., X.L. and Y.W., participated in the analysis. R.M., K.L., K.M.G. and S.B. drafted the manuscript. A.T., P.M., L.B., P.T. J.H. and S.B. performed the statistical analysis and prepared the tables/figures. K.L., K.M.G., C.C., G.B., H.I., N.K., J.H., C.E.C., C.P.C., Y.P., Y.L., Y.C., N.H. and P.C.C. participated in the study design and/or collected the samples/physiological measurements. K.L., K.M.G., C.C., M.H., P.D.G. conceived of the study, its design, and its coordination. All authors helped draft the manuscript, participated in manuscript editing and read/approved the final manuscript.

## Appendix A Supplementary Data

Supplementary material Image 1
