# Supplementary material for: ANRIL Promoter DNA Methylation: A Perinatal Marker for Later Adiposity
Source: eBioMedicine. 2017 Apr 26;19:60–72. doi: 10.1016/j.ebiom.2017.03.037 (PMC5440605; doi:10.1016/j.ebiom.2017.03.037)
Supplement: Supplementary file 1 — Supplementary material [file mmc1.docx]

**Supplementary Table 1. Association between conditional abdominal circumference growth Z-score from 34 weeks gestation to birth and umbilical cord *CDKN2A* methylation in the SWS.**

| ***CDKN2A* CpG** | **n** | **Abdominal circumference sex-specific LMS z-score growth from week 34 to birth** | |
| --- | --- | --- | --- |
|  |  | **Beta** | **P value** |
| **CpG 1** | 135 | 0.016 | 0.051 |
| **CpG 2** | 131 | 0.014 | 0.11 |
| **CpG 3** | 117 | 0.03 | **0.008**** |
| **CpG 4** | 154 | 0.018 | **0.034*** |
| **CpG 5** | 152 | 0.019 | **0.024*** |
| **CpG 6** | 152 | 0.015 | 0.077 |
| **CpG 7** | 151 | 0.018 | 0.071 |
| **CpG 8** | 139 | 0.018 | **0.046*** |
| **CpG 9** | 134 | 0.018 | 0.126 |

**Supplementary Table 2. Characteristics of the SWS study participants**

| **Characteristic** | **Group/number** | **% or median (inter-quartile range)** |
| --- | --- | --- |
| **Mother** |  |  |
| **Qualification level** | None | 0.9% |
|  | CSE | 9.9% |
|  | O levels | 27.0% |
|  | A levels | 26.4% |
|  | HND | 9.1% |
|  | Degree | 27.6% |
| **Social class** | Professional | 6.4% |
|  | Management/technical | 42.2% |
|  | Skilled non-manual | 33.2% |
|  | Skilled manual | 6.9% |
|  | Partly skilled | 10.4% |
|  | Unskilled | 0.9% |
| **Current smoker** | No | 77.0% |
|  | Yes | 23.0% |
| **Parity** | Primiparous | 47.7% |
|  | Multiparous | 52.3% |
| **Age at birth of child** | 352 | 30.7 (28.1 to 33.4) |
| **Early pregnancy BMI** | 279 | 24.8 (22.7 to 28.2) |
| **Offspring** |  |  |
| **Sex** | Male | 52.8% |
|  | Female | 47.2% |
| **Birthweight (kg)** | 349 | 3.4 (3.2 to 3.8) |
| **Gestation (wks.)** | 352 | 40.0 (39.0 to 40.9) |
| **Total fat (g) at birth** | 147 | 497.9 (364.3 to 644.3) |
| **Total fat (kg) at 4 years** | 283 | 4.0 (3.4 to 4.8) |
| **Total fat (kg) at 6 years** | 264 | 4.5 (3.6 to 6.0) |
| **%fat at birth** | 147 | 14.3 (11.6 to 17.3) |
| **%fat at 4 years** | 283 | 27.8 (24.3 to 32.5) |
| **%fat at 6 years** | 263 | 24.2 (19.9 to 29.7) |
| **Height (cm) at 4 years** | 350 | 103.9 (101.2 to 106.9) |
| **Height (cm) at 6 years** | 282 | 120 (116.7 to 124.3) |

**Supplementary Table 3.** Genes containing differentially methylated regions of interests (DMRs) identified from the MBD array using Fisher Exact tests, sorted by Fisher Exact test p-value.

| **Gene** | **Chr** | **p value** | **Gene** | **Chr** | **p value** |
| --- | --- | --- | --- | --- | --- |
| FEN1 | 11 | 1.00E-06 | SLC39A6 | 18 | 0.003518 |
| CCND1 | 11 | 1.00E-06 | BRSK2 | 11 | 0.003586 |
| GAPDH | 12 | 1.00E-06 | PRRC1 | 5 | 0.003859 |
| NGB | 14 | 1.00E-06 | C14orf142 | 14 | 0.003905 |
| GUSB | 7 | 1.00E-06 | APOL5 | 22 | 0.003905 |
| CCDC147 | 10 | 6.00E-06 | WWC2 | 4 | 0.004009 |
| ZNF17 | 19 | 6.00E-06 | DDX10 | 11 | 0.004046 |
| GATA6 | 18 | 2.40E-05 | DPF3 | 14 | 0.004774 |
| MPV17 | 19 | 2.90E-05 | GNG4 | 1 | 0.004802 |
| AKAP8 | 19 | 3.20E-05 | LAMA5 | 20 | 0.005051 |
| TNFRSF19 | 13 | 4.20E-05 | DSCR9 | 21 | 0.005272 |
| ABCA4 | 1 | 4.70E-05 | RNF43 | 17 | 0.005397 |
| TMEM116 | 12 | 5.50E-05 | DGKZ | 11 | 0.005776 |
| SETD4 | 21 | 5.60E-05 | TRIM13 | 13 | 0.006775 |
| SEZ6 | 17 | 6.50E-05 | FAM174B | 15 | 0.006871 |
| ZNF56 | 19 | 7.60E-05 | TOP2A | 17 | 0.007924 |
| APP | 21 | 9.30E-05 | NGFR | 17 | 0.007924 |
| CREBL2 | 12 | 0.000121 | C10orf107 | 10 | 0.009278 |
| ZSWIM1 | 20 | 0.000146 | PCNA | 20 | 0.010237 |
| SP1 | 12 | 0.000228 | PALB2 | 16 | 0.012353 |
| FBXO15 | 18 | 0.000281 | DHX34 | 19 | 0.012353 |
| OR4C11 | 11 | 0.000282 | CD1E | 1 | 0.013548 |
| C17orf58 | 17 | 0.000297 | HSF5 | 17 | 0.014521 |
| SAA1 | 11 | 0.00037 | INTS7 | 1 | 0.014585 |
| SIAH1 | 16 | 0.000379 | IFNK | 9 | 0.014585 |
| COPZ1 | 12 | 0.000479 | LOC100130987 | 11 | 0.014747 |
| LIN52 | 14 | 0.000655 | ZNF420 | 19 | 0.014747 |
| C1orf116 | 1 | 0.000813 | VN1R4 | 19 | 0.016052 |
| SLC6A4 | 17 | 0.000894 | OR5D18 | 11 | 0.0181 |
| ACTR6 | 12 | 0.000942 | TMEM109 | 11 | 0.018377 |
| MED16 | 19 | 0.000948 | CDKN2A | 9 | 0.018945 |
| C22orf39 | 22 | 0.000948 | HLA-C | 6 | 0.019681 |
| FYN | 6 | 0.001036 | LTBR | 12 | 0.020236 |
| MLEC | 12 | 0.001079 | TMEM61 | 1 | 0.022593 |
| ID1 | 20 | 0.001079 | MTNR1B | 11 | 0.02273 |
| AMZ2 | 17 | 0.00129 | CD40 | 20 | 0.025506 |
| FGF14 | 13 | 0.001357 | CLIC6 | 21 | 0.029338 |
| BACH1 | 21 | 0.001549 | MRPL16 | 11 | 0.032981 |
| EXD2 | 14 | 0.00176 | FAR1 | 11 | 0.03451 |
| CBLN4 | 20 | 0.001803 | PSMD11 | 17 | 0.036848 |
| MEG8 | 14 | 0.001841 | GNAS | 20 | 0.036848 |
| NDUFS8 | 11 | 0.002252 | CADM3 | 1 | 0.038332 |
| PCBP2 | 12 | 0.002439 | TMEM49 | 17 | 0.04337 |
| SNORD17 | 20 | 0.002526 | FGF14 | 13 | 0.045241 |
| ZNF664 | 12 | 0.00283 | KRTAP | 21 | 0.045241 |
| OR11H6 | 14 | 0.003012 | OAS1 | 12 | 0.04992 |
| OR4K5 | 14 | 0.003253 |  |  |  |

**Supplementary Table 4. Network and analysis using IPA Core Analysis.** The top 93 DMRs (all p ≤ 0.05) were analysed for enrichment within the same gene interaction networks.

| **Top networks** | **P** |
| --- | --- |
| DNA Replication, recombination and repair, cell death and survival, gene expression | 1.00E-41 |
| Gene expression, cell death and survival, embryonic development | 1.00E-25 |
| Cell morphology, cell-to-cell signalling and interaction, tissue development | 1.00E-12 |
| Cell cycle, cellular assembly and organization, cellular compromise | 0.01 |
| Organ morphology, cancer, endocrine system disorders | 0.01 |

**Supplementary Table 5. Surrogate Variable Analysis carried out on the BATMAN array results**

|  | | **DMR region** | | **Before Surrogate Variable Analysis** | | **After Surrogate Variable Analysis** | |
| --- | --- | --- | --- | --- | --- | --- | --- |
|  |  |  |  |  |  |  |  |
| **Gene** | **Chr** | **Start** | **End** | **β** | **p-value** | **β** | **p-value** |
| **CDKN2A** | 9 | 21993565 | 21993664 | -27.2 | 0.0028 | -32.0 | 0.0310 |
|  |  | 21993665 | 21993764 | -22.8 | 0.0025 | -25.2 | 0.0306 |

**Supplementart Table 6. Full unclustered associations between *CDKN2A* DMR methylation and percentage and total fat mass in the SWS cohort.** Associations between % and total fat mass at birth, 4 years and 6 years for *CDKN2A*. *** p = 0.05-0.01. ** p ≤ 0.01**; n=number of subjects, β=regression coefficient. Adjusted for sex, maternal age at birth and mode of delivery.

| **CDKN2A** | **4 yr. DXA: Percentage fat** | | | **4 yr. DXA: total fat** | | | **6 yr. DXA: Percentage fat** | | | **6 yr. DXA: total fat** | | |
| --- | --- | --- | --- | --- | --- | --- | --- | --- | --- | --- | --- | --- |
| **CpG** | **n** | **β** | **p-value** | **n** | **β** | **p-value** | **n** | **β** | **p-value** | **n** | **β** | **p-value** |
| **1** | 221 | -0.088 | **0.007**** | 221 | -0.004 | **0.007**** | 208 | -0.004 | **0.003**** | 209 | -0.005 | **0.017*** |
| **2** | 215 | -0.09 | **0.007**** | 215 | -0.005 | **0.003**** | 204 | -0.004 | **0.003**** | 205 | -0.005 | **0.011*** |
| **3** | 192 | -0.074 | 0.111 | 192 | -0.005 | **0.024*** | 184 | -0.005 | **0.007**** | 184 | -0.007 | **0.014*** |
| **4** | 247 | -0.092 | **0.007**** | 247 | -0.005 | **0.001**** | 231 | -0.004 | **0.005**** | 232 | -0.007 | **0.001**** |
| **5** | 246 | -0.054 | 0.101 | 246 | -0.004 | **0.018*** | 230 | -0.002 | 0.101 | 231 | -0.005 | **0.022*** |
| **6** | 246 | -0.06 | 0.06 | 246 | -0.004 | **0.005**** | 230 | -0.004 | **0.012*** | 231 | -0.007 | **0.002**** |
| **7** | 244 | -0.076 | **0.04*** | 244 | -0.004 | **0.015*** | 228 | -0.003 | 0.042 | 229 | -0.006 | **0.016*** |
| **8** | 228 | -0.057 | 0.096 | 228 | -0.004 | **0.017*** | 212 | -0.002 | 0.236 | 213 | -0.005 | **0.048*** |
| **9** | 218 | -0.074 | 0.089 | 218 | -0.005 | **0.011*** | 203 | -0.003 | 0.122 | 204 | -0.007 | **0.022*** |

**Supplementary Table 7. GUSTO subject characteristics**

| **Mother** | **Mean (SD) or %** | **n** |
| --- | --- | --- |
| **Household monthly income (SGD)** |  | 373 |
| 0-1999 | 17.7 |  |
| 2000-5999 | 63.8 |  |
| ≥6000 | 18.5 |  |
| **Maternal highest education %** |  | 392 |
| ITE_NTC | 12 |  |
| Primary | 5.4 |  |
| Secondary | 32.1 |  |
| GCE A level | 25 |  |
| University | 22.7 |  |
| Others | 2.8 |  |
| **Age at birth, years** | 30.5 (5.3) | 399 |
| **Smoker** | 3.7 | 397 |
| **BMI at 26weeks pregnancy** | 26.9 (5.0) | 394 |
| **Infant** |  |  |
| **Sex(%)** |  | 399 |
| Male | 53.1 |  |
| Female | 46.9 |  |
| Parity |  | 399 |
| Primiparous | 40.4 |  |
| Multiparous | 59.6 |  |
| Birth weight, kg | 3.1 (0.4) | 399 |
| Gestational age, weeks | 38.7 (1.2) | 399 |
| Triceps skinfold thickness at 7-days, mm | 6.4 (1.5) | 344 |
| Subscapular skinfold thickness at 7-days, mm | 5.9 (1.5) | 344 |
| Ponderal index age 18-months kg/m3 | 19 (2.0) | 299 |

**Supplementary Table 8. Associations between umbilical cord *CDKN2A* methylation and ponderal index and subscapular skinfold thickness in the GUSTO cohort at day 7 and 18 months.** Adjusted for: gender, ethnicity and cell type. n=number of subjects, b=regression coefficient.

|  | | **Ponderal index** (kg/m^3^) | | | **Subscapular skinfold thickness** (mm) | | |
| --- | --- | --- | --- | --- | --- | --- | --- |
| **cluster** | **Timepoint** | **n** | **β** | **p-value** | **n** | **β** | **p-value** |
| **1-2** | **day 7** | 242 | -1.58 | 0.718 | 242 | -0.926 | 0.747 |
|  | **18 month** | 215 | -6.99 | 0.061 | 205 | -2.661 | 0.349 |
| **3** | **day 7** | 217 | 0.27 | 0.960 | 217 | -9.638 | **0.004*** |
|  | **18 month** | 197 | -6.61 | 0.140 | 187 | -4.112 | 0.202 |
| **4-8** | **day 7** | 282 | 2.17 | 0.363 | 282 | -2.324 | 0.125 |
|  | **18 month** | 252 | -2.13 | 0.262 | 236 | -0.933 | 0.509 |
| **9** | **day 7** | 204 | -3.82 | 0.387 | 204 | -4.465 | 0.085 |
|  | **18 month** | 183 | -10.61 | **0.005*** | 165 | -5.822 | 0.046 |

**Supplementary Table 9. Gene expression correlates with adiposity measurements in the GUSTO cohort.** Adiposity measurements at day 7 and 18 months from infants in the GUSTO cohort were correlated to expression levels (dCt) of ANRIL linear, ANRIL unspliced, ANRIL circular, p14^ARF^ and p16^INK4a^ transcript levels, controlling for gender and ethnicity. n=number of subjects, b=regression coefficient.

|  | | **Ponderal index** (kg/m^3^) | | | **Triceps skinfold thickness** (mm) | | | **Subscapular skinfold thickness** (mm) | | |
| --- | --- | --- | --- | --- | --- | --- | --- | --- | --- | --- |
| **Transcript** | **Timepoint** | **n** | **β** | **p-value** | **n** | **β** | **p-value** | **n** | **β** | **p-value** |
| **ANRIL (Linear)** | **day 7** | 143 | -0.03 | 0.24 | 143 | 0.19 | 0.22 | 143 | 0.12 | 0.47 |
|  | **18 month** | 148 | 0.01 | 0.56 | 144 | 0.19 | 0.31 | 140 | 0.11 | 0.42 |
| **ANRIL (Circular)** | **day 7** | 94 | 0.01 | 0.69 | 94 | 0.12 | **0.02** | 94 | 0.12 | **0.05** |
|  | **18 month** | 99 | 0.01 | 0.86 | 93 | -0.09 | 0.12 | 92 | -0.05 | 0.24 |
| **ANRIL (Unspliced)** | **day 7** | 143 | 0.01 | 0.91 | 143 | 0.51 | **0.01** | 143 | 0.63 | **0.01** |
|  | **18 month** | 148 | -0.01 | 0.76 | 144 | 0.34 | 0.11 | 140 | 0.23 | 0.15 |
| **p14ARF** | **day 7** | 143 | -0.04 | **0.04** | 143 | 0.03 | 0.81 | 143 | 0.06 | 0.68 |
|  | **18 month** | 148 | 0.01 | 0.82 | 144 | 0.15 | 0.31 | 141 | 0.18 | 0.13 |
| **p16INK4a** | **day 7** | 110 | -0.01 | 0.43 | 110 | 0.06 | 0.52 | 110 | 0.10 | 0.34 |
|  | **18 month** | 122 | 0.01 | 0.34 | 116 | 0.23 | **0.04** | 113 | 0.20 | **0.02** |

**Supplementary Table 10. Characteristics of the RAINE study participants.**

| **Characteristic** | **Group/number** | **% or median (inter-quartile range)** |
| --- | --- | --- |
| **Mother** |  |  |
| **Qualification level** | High school only | 51.60% |
|  | Trade Certificate or apprenticeship | 8.40% |
|  | Professional Registration | 8.80% |
|  | Diploma | 16.00% |
|  | University Degree | 10.00% |
|  | Other | 5.20% |
| **Smoker During Pregnancy** | No | 82.80% |
|  | Yes | 17.20% |
| **Parity** | Primiparous | 49.40% |
|  | Multiparous | 50.60% |
| **Age at birth of child (years)** | 2804 | 28 (23 to 32) |
| **Early pregnancy BMI** | 2803 | 21.3 (19.6 to 23.7) |
| **Offspring** |  |  |
| **Sex** | Male | 50.70% |
|  | Female | 49.30% |
| **Birthweight (g)** | 2858 | 3345 (2984 to 3660) |
| **Birth Length (cm)** | 2826 | 49 (47.5 to 50.5) |
| **Gestation (wks.)** | 2862 | 39.5 (38.2 to 40.7) |
| **Weight (kg) at 17 years** | 1251 | 65.4 (58.2 to 74.6) |
| **Height (cm) at 17 years** | 1251 | 172 (165 to 179) |
| **BMI (kg/m2) at 17 years** | 1251 | 22.06 (19.96 to 24.33) |
| **Abdominal skinfold thickness (mm) at 17 years** | 1155 | 19.9 (12.6 to 27.5) |
| **Triceps Skinfold Thickness (mm) at 17 years** | 1170 | 14.0(9.0 to 19.5) |

**Supplementary Table 11A. Association between cellular type and BMI in the RAINE cohort. N = 894**

| **cell sub type** | **association with BMI** | |
| --- | --- | --- |
|  | **Pearson Correlation** | **Sig. (2-tailed)** |
| **CD8T** | -0.098 | 0.003 |
| **CD4T** | -0.082 | 0.015 |
| **NK** | -0.079 | 0.018 |
| **B cell** | -0.079 | 0.018 |
| **Mono** | 0.043 | 0.201 |
| **Gran** | 0.160 | 0.001 |

**Supplementary Table 11B. Association between *CDKN2A* CpG methylation and cellular type in the RAINE cohort**

| **CpG cluster** | **N** | **CD8T** | | **CD4T** | | **NK** | | **B cell** | | **Mono** | | **Gran** | |
| --- | --- | --- | --- | --- | --- | --- | --- | --- | --- | --- | --- | --- | --- |
|  |  | **Pearson Correlation** | **Sig. (2-tailed)** | **Pearson Correlation** | **Sig. (2-tailed)** | **Pearson Correlation** | **Sig. (2-tailed)** | **Pearson Correlation** | **Sig. (2-tailed)** | **Pearson Correlation** | **Sig. (2-tailed)** | **Pearson Correlation** | **Sig. (2-tailed)** |
| **1-2** | 1013 | 0.359 | <2.2e-16 | 0.300 | < 2.2e-16 | 0.252 | 4.44e-16 | 0.085 | 0.0104 | -0.229 | 2.41e-12 | -0.452 | < 2.2e-16 |
| **3** | 969 | 0.443 | <2.2e-16 | 0.411 | < 2.2e-16 | 0.253 | 1.33e-15 | 0.142 | 9.50e-06 | -0.319 | < 2.2e-16 | -0.562 | < 2.2e-16 |
| **4-8** | 986 | 0.618 | <2.2e-16 | 0.496 | < 2.2e-16 | 0.420 | < 2.2e-16 | 0.185 | 8.90e-09 | -0.394 | < 2.2e-16 | -0.762 | < 2.2e-16 |
| **9** | 1009 | 0.591 | <2.2e-16 | 0.621 | < 2.2e-16 | 0.364 | < 2.2e-16 | 0.243 | 8.88e-15 | -0.441 | < 2.2e-16 | -0.824 | < 2.2e-16 |

**Supplementary Table 11C. Variance Inflation Factors for *CDKN2A* CpG methylation values and cellular type in the RAINE cohort**

|  | **CD8+ T cells** | **CD4+ T cells** | **NK cells** | **B-cell** | **Monocytes** | **granulocytes** |
| --- | --- | --- | --- | --- | --- | --- |
| **CpG 1** | 25.2 | 61.3 | 27.6 | 14.3 | 11.4 | 110.5 |
| **CpG 2** | 25.4 | 61.1 | 28.3 | 14.5 | 11.6 | 112.1 |
| **CpG 3** | 25.6 | 61.5 | 27.9 | 14.6 | 11.6 | 112.8 |
| **CpG 4** | 25.0 | 61.2 | 30.3 | 14.7 | 11.3 | 113.7 |
| **CpG 5** | 25.2 | 61.7 | 29.9 | 14.5 | 11.4 | 115.7 |
| **CpG 6** | 24.9 | 61.3 | 29.8 | 14.5 | 11.4 | 114.8 |
| **CpG 7** | 25.3 | 62.9 | 30.3 | 15.1 | 11.8 | 116.9 |
| **CpG 8** | 24.8 | 61.4 | 30.1 | 14.7 | 11.4 | 114.1 |
| **CpG 9** | 24.7 | 61.1 | 29.6 | 14.2 | 11.3 | 112.9 |

**Supplementary Table 12.** **Characteristics of the BIOCLAIMS study participants.** Participants are grouped by % fat mass (lean/obese). Median and interquartile ranges or % are shown as appropriate.

| **Measurements** | **% or median (5th,95th percentile) for BIOCLAIMS Cohort (adipose tissue) n=51** | |
| --- | --- | --- |
|  |  |  |
|  | **Lean according to % fat mass** | **Obese according to % fat mass** |
|  |  |  |
| Age at visit | 25.60 (18.93 to 57.11) | 45.75 (22.82 to 62.3) |
| Weight (kg) | 59.80 (47.16 to 98.09) | 96.55 (66.01 to 125.73) |
| Height (M) | 1.65 (1.48 to 1.91) | 1.65 (1.53 to 1.85) |
| BMI | 22.50 (18.11 to 26.82) | 34.28 (23.63 to 40.09) |
| Waist, cm | 74.20 (65.70 to 96.09) | 105.85 (90.86 to 127.85) |
| Hip, cm | 94.40 (76.86 to 103.92) | 117.00 (97.42 to 134.02) |
| Percentage fat | 24.50 (12.24 to 31.45) | 42.65 (26.80 to 50.95) |
| Fatmass, kg | 14.60 (8.72 to 20.79) | 36.90 (22.54 to 56.90) |
| Total body water, kg | 31.10 (26.45 to 57.29) | 38.60 (30.27 to 62.45) |
| Male/Female | 19.05%/80.95% | 23.33%/76.67% |
| Lean mass, kg | 42.40 (36.16 to 78.26) | 52.70 (41.38 to 85.28) |

**Supplementary Table 13. Observed DNA methylation ranges for CpG dinucleotides quantified by Pyrosequencing.** Values are provided as percentages for pyrosequencing assays covering BATMAN-identified DMRs associated with *CDKN2A* (SWS/GUSTO)

|  | **CDKN2A** | | | |
| --- | --- | --- | --- | --- |
|  | **Median (5th, 95th percentile)** | | **Base pairs from TSS** |  |
| **CpG** | **SWS** | **GUSTO** | **ANRIL** | **Coordinates (Hg19)** |
| 1 | 71.8 (48.7, 81.3) | 81.8 (70.8, 87.1) | -1069 | chr9: 21993721 |
| 2 | 67.3 (44.8, 77.9) | 76.7 (67.1, 81.6) | -1093 | chr9: 21993697 |
| 3 | 54.8 (38.4, 62.6) | 65.8 (58.6, 70.0) | -1096 | chr9: 21993694 |
| 4 | 70.7 (50.6, 80.5) | 77.6 (59.9, 86.1) | -1136 | chr9: 21993654 |
| 5 | 58.3 (36.7, 69.6) | 67.5 (51.5, 75.7) | -1145 | chr9: 21993645 |
| 6 | 70.2 (49.6, 79.8) | 77.1 (58.7, 83.6) | -1152 | chr9: 21993638 |
| 7 | 58.2 (42.2, 68.0) | 65.9 (48.7, 73.2) | -1161 | chr9: 21993629 |
| 8 | 73.0 (55.3, 81.6) | 81.3 (62.6, 87.5) | -1187 | chr9: 21993603 |
| 9 | 60.4 (46.2, 67.6) | 76.4 (67.8, 79.8) | -1207 | chr9: 21993583 |

**Supplementary Table 14.** Primers used for pyrosequencing and real-time PCR, and in the cloning of the ANRIL promoter region.

| **Pyrosequencing Primer** | **Sequence** |
| --- | --- |
| CDKN2A 1-3 F | AGTAGGAAAGGTGTATTTTAAGTATATTT |
| CDKN2A 1-3 S | AGAATTATTGTTAATTATTTAAGTT |
| CDKN2A 4-9 F | TGGGGAGAATTATTGTTAATTATTTAAGTT |
| CDKN2A 4-9 S | TAGGAGAGTGGAGGA |
| CDKN2A 8-9 S | GTAGGTAGAGATTTTTTGAAATGT |
| CDKN2A 1-3 R Bio | TATCTCACCAATCCTCCACTCTCCTAAA |
| CDKN2A 4-9 R Bio | AAAAACCCATTTCCCTATTAACTACA |
| **Real-time primer** | **Sequence** |
| ANRIL unspliced F | CAGTGGCTTCCTGTTCATGC |
| ANRIL unspliced R | GGGCTTGACGTCTGATCTGT |
| ANRIL F1 | TGAAAAACACACATCAAAGGAG |
| ANRIL R1 | GATTCCACCACACCTAACAG |
| P14ARF F1 | CCCTCGTGCTGATGCTACTGAG |
| P14ARF R1 | GTGAGAGTGGCGGGGTCGGC |
| P16INK4a F1 | CGAATAGTTACGGTCGGAGGC |
| P16INK4a R1 | TCGGGTGAGAGTGGCGGGGTC |
| **Cloning Primer** | **sequence** |
| ANRIL F1 | TTGGGTACCACCTCTAACTCACAAAGAAAGC |
| ANRIL R1 | GCCAAGCTTTGGGAATGACTAAGACACAC |
| ANRIL F2 | GAGAAGCTTCCCAGGATATTCGGGACTCA |
| ANRIL R2 | TTACCATGGTGTCAGGTGACGGATGTAGC |
| **EMSA Primer** | **sequence** |
| CDK EMSA 1F | TTTCTGGAGGCGGCCTTTTTTC |
| CDK EMSA 2,3F | CCCCAGCCTCCCGGCGGGGTCACCC |
| CDK EMSA 4-7F | GAGTGGAGGACCCGTGAGATACGGGGCACGCAGGCAGCGACTTCCTGAA |
| CDK EMSA 8, 9F | CTAACAAGGATCGTAGGATCAGTTACTGCTGCGAG |
| CDK EMSA 1R | GAAAAAAGGCCGCCTCCAGAAA |
| CDK EMSA 2,3R | GGGTGACCCCGCCGGGAGGCTGGGG |
| CDK EMSA 4-7R | TTCAGGAAGTCGCTGCCTGCGTGCCCCGTATCTCACGGGTCCTCCACTC |
| CDK EMSA 8, 9R | CTCGCAGCAGTAACTGATCCTACGATCCTTGTTAG |
| ER alpha F | GATTCCTGAAGGTCAAGGTCACCAGAGCTTGGCCTG |
| ER alpha R | CAGGCCAAGCTCTGGTGACCTTGACCTTCAGGAATC |

**Supplementary Figure 1. Association between conditional abdominal circumference growth Z-score from 34 weeks gestation to birth and umbilical cord CpG3 methylation in the SWS.**

**Supplementary Figure 2. CpG Clustering.** Spearman correlation of methylation levels at CpGs 1-9 within the ANRIL promoter. Four distinct clusters are defined: 1-2, 3, 4-8 and 9. (A) correlations in the RAINE cohort. (B) correlations in the BIOCLAIMS cohort.

(B) BIOCLAIMS cohort correlations

(A) RAINE cohort correlations
